# Ethyl 2-[4-(di­methyl­amino)­phen­yl]-1-phenyl-1*H*-benzimidazole-5-carboxyl­ate

**DOI:** 10.1107/S1600536813012440

**Published:** 2013-05-15

**Authors:** Keng Yoon Yeong, Mohamed Ashraf Ali, Tan Soo Choon, Mohd Mustaqim Rosli, Ibrahim Abdul Razak

**Affiliations:** a Institute for Research in Molecular Medicine, Universiti Sains Malaysia, 11800 USM, Penang, Malaysia; b X-ray Crystallography Unit, School of Physics, Universiti Sains Malaysia, 11800 USM, Penang, Malaysia

## Abstract

In the title compound, C_24_H_23_N_3_O_2_, the benzimidazole ring system makes dihedral angles of 7.28 (5) and 67.17 (5)°, respectively, with the planes of the benzene and phenyl rings, which in turn make a dihedral angle of 69.77 (6)°. In the crystal, mol­ecules are connected by C—H⋯N and C—H⋯O inter­actions, forming a layer parallel to the *bc* plane. A π–π inter­action, with a centroid–centroid distance of 3.656 (1) Å, is observed in the layer.

## Related literature

For applications of benzimidazole compounds, see: Rao *et al.* (2002 ▶); Thakurdesai *et al.* (2007 ▶); McKellar & Scott (1990 ▶). For a related structure, see: Yoon *et al.* (2012 ▶). For the stability of the temperature controller used in the data collection, see: Cosier & Glazer (1986 ▶).
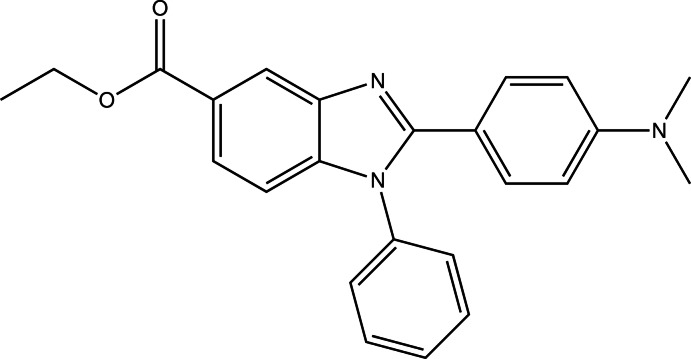


## Experimental

### 

#### Crystal data

C_24_H_23_N_3_O_2_
*M* *_r_* = 385.45
Triclinic, 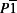

*a* = 8.7196 (1) Å
*b* = 10.4133 (2) Å
*c* = 11.3658 (2) Å
α = 79.312 (1)°
β = 74.393 (1)°
γ = 89.781 (1)°
*V* = 975.56 (3) Å^3^
*Z* = 2
Mo *K*α radiation
μ = 0.09 mm^−1^
*T* = 100 K
0.36 × 0.25 × 0.25 mm

#### Data collection

Bruker SMART APEXII CCD area-detector diffractometer
Absorption correction: multi-scan (*SADABS*; Bruker, 2009 ▶) *T*_min_ = 0.970, *T*_max_ = 0.980
21016 measured reflections
5777 independent reflections
4602 reflections with *I* > 2σ(*I*)
*R*_int_ = 0.029

#### Refinement

*R*[*F* ^2^ > 2σ(*F* ^2^)] = 0.050
*wR*(*F* ^2^) = 0.125
*S* = 1.03
5777 reflections
265 parameters
H-atom parameters constrained
Δρ_max_ = 0.38 e Å^−3^
Δρ_min_ = −0.30 e Å^−3^

### 

Data collection: *APEX2* (Bruker, 2009 ▶); cell refinement: *SAINT* (Bruker, 2009 ▶); data reduction: *SAINT*; program(s) used to solve structure: *SHELXTL* (Sheldrick, 2008 ▶); program(s) used to refine structure: *SHELXTL*; molecular graphics: *SHELXTL*; software used to prepare material for publication: *SHELXTL* and *PLATON* (Spek, 2009 ▶).

## Supplementary Material

Crystal structure: contains datablock(s) I, global. DOI: 10.1107/S1600536813012440/is5269sup1.cif

Structure factors: contains datablock(s) I. DOI: 10.1107/S1600536813012440/is5269Isup2.hkl

Supplementary material file. DOI: 10.1107/S1600536813012440/is5269Isup3.cml

Additional supplementary materials:  crystallographic information; 3D view; checkCIF report

## Figures and Tables

**Table 1:** Hydrogen-bond geometry (Å, °)

*D*—H⋯*A*	*D*—H	H⋯*A*	*D*⋯*A*	*D*—H⋯*A*
C15—H15*A*⋯N2^i^	0.95	2.57	3.4868 (16)	164
C18—H18*A*⋯O2^ii^	0.95	2.50	3.2217 (17)	133
C19—H19*A*⋯O2^iii^	0.95	2.38	3.3209 (16)	170

Symmetry codes: (i) 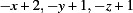; (ii) 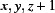; (iii) 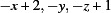.

**Fig. 1.**
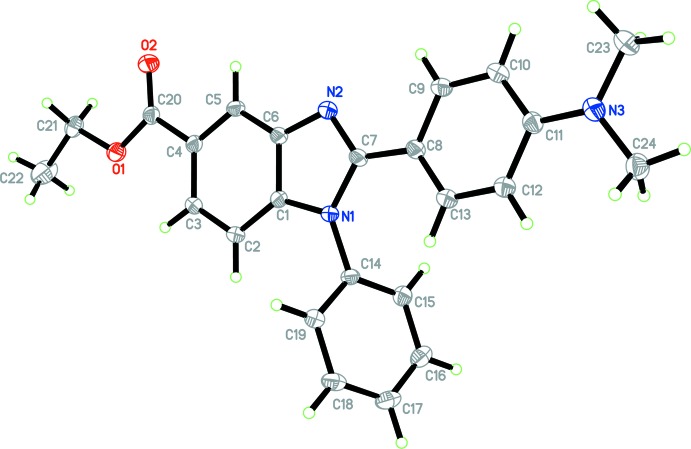
The molecular structure of the title compound, showing 50% probability displacement ellipsoids.

**Fig. 2.**
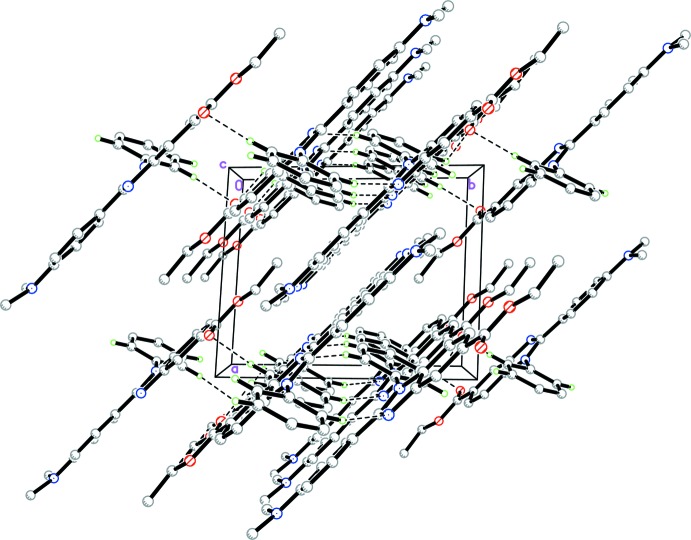
A crystal packing view of the title compound. Dashed lines indicate hydrogen bonds. H atoms not involved in the hydrogen bonds have been omitted for clarity.

## supplementary crystallographic information

### Comment

The synthesis of benzimidazole derivatives is an active area of research in medicinal chemistry. Benzimidazoles are a class of bioactive heterocyclic compounds which exhibit a wide range of activities such as anti-HIV (Rao *et al.*, 2002), anti-inflammatory (Thakurdesai *et al.*, 2007) and anthelmintics (McKellar & Scott, 1990). As part of our ongoing structural studies on benzimidazole derivatives (Yoon *et al.*, 2012), we now report the structure of the title compound.

In the title compound (Fig. 1), the benzimidazole ring (N1/N2/C1–C7) is planar with a maximum deviation of 0.025 (1) Å for atom N1. It makes dihedral angles of 7.28 (5) and 67.17 (5)°, respectively, with the benzene (C8–C13) and phenyl (C14–C19) rings, and these two rings make a dihedral angle of 69.77 (6)° with each other.

In the crystal (Fig. 2), the molecules are connected by intermolecular C15—H15A···N2^i^, C18—H18A···O2^ii^ and C19—H19A···O2^iii^ interactions (Table 1) to form two-dimensional layers parallel to the *bc*-plane. A π–π interaction between the benzene rings of C1–C6 and C8–C13 also contributes in stabilizing the crystal structure with their centroid distances of 3.656 (1) Å (2 - *x*, 1 - *y*, 1 - *z*).

### Experimental

Ethyl 3-amino-4-(penylamino)benzoate (0.84 mmol) and sodium metabisulfite adduct of 4-dimethylamino benzaldehyde (1.68 mmol) were dissolved in DMF. The reaction mixture was reflux at 130 °C for 2 hrs. After completion, the reaction mixture was diluted in Ethyl acetate (20 ml) and washed with water (20 ml). The organic layer was collected, dried over Na_2_SO_4_ and the evaporated *in vacuo* to yield the product. The product was recrystallized from Ethyl acetate.

### Refinement

All the H atoms were positioned geometrically and refined using a riding model with with C—H = 0.95–0.99 Å and *U*_iso_(H) = 1.2*U*_eq_(C) or 1.5*U*_eq_(C) for methyl H atoms. A rotating group model was applied to the methyl group.

### Crystal data

**Table:**

C_24_H_23_N_3_O_2_	*Z* = 2
*M**_r_* = 385.45	*F*(000) = 408
Triclinic, *P*1	*D*_x_ = 1.312 Mg m^−^^3^
Hall symbol: -P 1	Mo *K*α radiation, λ = 0.71073 Å
*a* = 8.7196 (1) Å	Cell parameters from 7512 reflections
*b* = 10.4133 (2) Å	θ = 2.5–30.2°
*c* = 11.3658 (2) Å	µ = 0.09 mm^−^^1^
α = 79.312 (1)°	*T* = 100 K
β = 74.393 (1)°	Block, yellow
γ = 89.781 (1)°	0.36 × 0.25 × 0.25 mm
*V* = 975.56 (3) Å^3^	

### Data collection

**Table:**

Bruker SMART APEXII CCD area-detector diffractometer	5777 independent reflections
Radiation source: fine-focus sealed tube	4602 reflections with *I* > 2σ(*I*)
Graphite monochromator	*R*_int_ = 0.029
φ and ω scans	θ_max_ = 30.2°, θ_min_ = 1.9°
Absorption correction: multi-scan (*SADABS*; Bruker, 2009)	*h* = −12→12
*T*_min_ = 0.970, *T*_max_ = 0.980	*k* = −14→14
21016 measured reflections	*l* = −15→16

### Refinement

**Table:**

Refinement on *F*^2^	Primary atom site location: structure-invariant direct methods
Least-squares matrix: full	Secondary atom site location: difference Fourier map
*R*[*F*^2^ > 2σ(*F*^2^)] = 0.050	Hydrogen site location: inferred from neighbouring sites
*wR*(*F*^2^) = 0.125	H-atom parameters constrained
*S* = 1.03	*w* = 1/[σ^2^(*F*_o_^2^) + (0.0552*P*)^2^ + 0.401*P*] where *P* = (*F*_o_^2^ + 2*F*_c_^2^)/3
5777 reflections	(Δ/σ)_max_ = 0.001
265 parameters	Δρ_max_ = 0.38 e Å^−^^3^
0 restraints	Δρ_min_ = −0.30 e Å^−^^3^

### Special details

**Table:**

Experimental. The crystal was placed in the cold stream of an Oxford Cryosystems Cobra open-flow nitrogen cryostat (Cosier & Glazer, 1986) operating at 100.0 (1) K.
Geometry. All e.s.d.'s (except the e.s.d. in the dihedral angle between two l.s. planes) are estimated using the full covariance matrix. The cell e.s.d.'s are taken into account individually in the estimation of e.s.d.'s in distances, angles and torsion angles; correlations between e.s.d.'s in cell parameters are only used when they are defined by crystal symmetry. An approximate (isotropic) treatment of cell e.s.d.'s is used for estimating e.s.d.'s involving l.s. planes.
Refinement. Refinement of *F*^2^ against ALL reflections. The weighted *R*-factor *wR* and goodness of fit *S* are based on *F*^2^, conventional *R*-factors *R* are based on *F*, with *F* set to zero for negative *F*^2^. The threshold expression of *F*^2^ > σ(*F*^2^) is used only for calculating *R*-factors(gt) *etc*. and is not relevant to the choice of reflections for refinement. *R*-factors based on *F*^2^ are statistically about twice as large as those based on *F*, and *R*- factors based on ALL data will be even larger.

### Fractional atomic coordinates and isotropic or equivalent isotropic displacement parameters (Å^2^)

**Table:**

	*x*	*y*	*z*	*U*_iso_*/*U*_eq_	
O1	1.34962 (11)	−0.05308 (9)	0.29082 (8)	0.02242 (19)	
O2	1.18718 (12)	0.04740 (9)	0.18312 (9)	0.0266 (2)	
N1	0.98249 (12)	0.30045 (9)	0.65882 (9)	0.0171 (2)	
N2	0.88283 (12)	0.33970 (10)	0.49238 (9)	0.0181 (2)	
N3	0.41760 (14)	0.72466 (11)	0.79245 (11)	0.0251 (2)	
C1	1.05977 (14)	0.22039 (11)	0.57924 (11)	0.0166 (2)	
C2	1.17590 (15)	0.12968 (11)	0.58860 (11)	0.0191 (2)	
H2A	1.2151	0.1116	0.6600	0.023*	
C3	1.23128 (15)	0.06738 (11)	0.48914 (11)	0.0189 (2)	
H3A	1.3098	0.0043	0.4923	0.023*	
C4	1.17363 (14)	0.09557 (11)	0.38285 (11)	0.0171 (2)	
C5	1.05674 (14)	0.18556 (11)	0.37516 (11)	0.0173 (2)	
H5A	1.0180	0.2040	0.3036	0.021*	
C6	0.99820 (14)	0.24776 (11)	0.47532 (11)	0.0167 (2)	
C7	0.87548 (14)	0.37009 (11)	0.60175 (11)	0.0171 (2)	
C8	0.76152 (14)	0.46126 (11)	0.65582 (11)	0.0178 (2)	
C9	0.67109 (15)	0.52985 (12)	0.58132 (12)	0.0205 (2)	
H9A	0.6880	0.5167	0.4984	0.025*	
C10	0.55857 (15)	0.61573 (12)	0.62502 (12)	0.0216 (2)	
H10A	0.5000	0.6602	0.5718	0.026*	
C11	0.52922 (14)	0.63840 (11)	0.74735 (12)	0.0192 (2)	
C12	0.61759 (14)	0.56812 (12)	0.82302 (12)	0.0197 (2)	
H12A	0.5998	0.5801	0.9064	0.024*	
C13	0.72998 (14)	0.48187 (12)	0.77812 (11)	0.0193 (2)	
H13A	0.7870	0.4356	0.8317	0.023*	
C14	1.02675 (14)	0.31301 (11)	0.76961 (11)	0.0169 (2)	
C15	1.10245 (14)	0.42822 (12)	0.77495 (12)	0.0199 (2)	
H15A	1.1247	0.4994	0.7062	0.024*	
C16	1.14503 (15)	0.43744 (13)	0.88256 (12)	0.0234 (3)	
H16A	1.1955	0.5160	0.8879	0.028*	
C17	1.11435 (16)	0.33248 (14)	0.98248 (12)	0.0251 (3)	
H17A	1.1432	0.3398	1.0559	0.030*	
C18	1.04162 (15)	0.21708 (13)	0.97494 (12)	0.0234 (3)	
H18A	1.0224	0.1450	1.0427	0.028*	
C19	0.99673 (15)	0.20678 (12)	0.86827 (11)	0.0195 (2)	
H19A	0.9462	0.1282	0.8630	0.023*	
C20	1.23411 (14)	0.02962 (11)	0.27581 (11)	0.0186 (2)	
C21	1.40780 (16)	−0.12580 (13)	0.19169 (12)	0.0237 (3)	
H21A	1.4474	−0.0649	0.1109	0.028*	
H21B	1.3210	−0.1834	0.1862	0.028*	
C22	1.53955 (19)	−0.20550 (18)	0.22155 (17)	0.0406 (4)	
H22A	1.5714	−0.2651	0.1628	0.061*	
H22B	1.5030	−0.2564	0.3065	0.061*	
H22C	1.6309	−0.1474	0.2150	0.061*	
C23	0.34150 (17)	0.80634 (14)	0.70882 (14)	0.0289 (3)	
H23A	0.2964	0.7515	0.6638	0.043*	
H23B	0.4205	0.8701	0.6492	0.043*	
H23C	0.2562	0.8526	0.7569	0.043*	
C24	0.41620 (17)	0.76536 (13)	0.90791 (13)	0.0260 (3)	
H24A	0.3841	0.6904	0.9770	0.039*	
H24B	0.3404	0.8344	0.9219	0.039*	
H24C	0.5231	0.7988	0.9029	0.039*	

### Atomic displacement parameters (Å^2^)

**Table:**

	*U*^11^	*U*^22^	*U*^33^	*U*^12^	*U*^13^	*U*^23^
O1	0.0267 (5)	0.0233 (4)	0.0198 (4)	0.0066 (4)	−0.0075 (4)	−0.0088 (3)
O2	0.0379 (5)	0.0266 (5)	0.0194 (5)	0.0062 (4)	−0.0131 (4)	−0.0072 (4)
N1	0.0224 (5)	0.0161 (5)	0.0155 (5)	0.0039 (4)	−0.0093 (4)	−0.0038 (4)
N2	0.0211 (5)	0.0175 (5)	0.0164 (5)	0.0018 (4)	−0.0070 (4)	−0.0023 (4)
N3	0.0284 (6)	0.0242 (5)	0.0256 (6)	0.0092 (4)	−0.0107 (5)	−0.0074 (4)
C1	0.0210 (5)	0.0147 (5)	0.0148 (5)	0.0002 (4)	−0.0062 (4)	−0.0031 (4)
C2	0.0247 (6)	0.0177 (5)	0.0174 (5)	0.0034 (4)	−0.0105 (5)	−0.0028 (4)
C3	0.0237 (6)	0.0164 (5)	0.0183 (6)	0.0035 (4)	−0.0085 (5)	−0.0033 (4)
C4	0.0214 (5)	0.0144 (5)	0.0156 (5)	−0.0008 (4)	−0.0056 (4)	−0.0022 (4)
C5	0.0216 (5)	0.0173 (5)	0.0143 (5)	−0.0005 (4)	−0.0076 (4)	−0.0019 (4)
C6	0.0194 (5)	0.0146 (5)	0.0166 (5)	−0.0001 (4)	−0.0073 (4)	−0.0011 (4)
C7	0.0200 (5)	0.0145 (5)	0.0169 (5)	0.0005 (4)	−0.0072 (4)	−0.0001 (4)
C8	0.0197 (5)	0.0159 (5)	0.0179 (5)	0.0007 (4)	−0.0067 (4)	−0.0012 (4)
C9	0.0240 (6)	0.0217 (6)	0.0173 (6)	0.0036 (5)	−0.0087 (5)	−0.0034 (4)
C10	0.0232 (6)	0.0214 (6)	0.0219 (6)	0.0045 (5)	−0.0102 (5)	−0.0020 (5)
C11	0.0190 (5)	0.0167 (5)	0.0218 (6)	0.0010 (4)	−0.0060 (5)	−0.0026 (4)
C12	0.0215 (6)	0.0199 (6)	0.0180 (6)	0.0018 (4)	−0.0064 (4)	−0.0028 (4)
C13	0.0217 (6)	0.0190 (5)	0.0174 (5)	0.0021 (4)	−0.0072 (4)	−0.0013 (4)
C14	0.0195 (5)	0.0194 (5)	0.0141 (5)	0.0046 (4)	−0.0074 (4)	−0.0049 (4)
C15	0.0213 (6)	0.0193 (6)	0.0195 (6)	0.0023 (4)	−0.0053 (4)	−0.0050 (4)
C16	0.0223 (6)	0.0270 (6)	0.0240 (6)	0.0006 (5)	−0.0076 (5)	−0.0108 (5)
C17	0.0244 (6)	0.0356 (7)	0.0193 (6)	0.0040 (5)	−0.0101 (5)	−0.0095 (5)
C18	0.0254 (6)	0.0286 (6)	0.0166 (6)	0.0041 (5)	−0.0086 (5)	−0.0010 (5)
C19	0.0220 (6)	0.0187 (5)	0.0191 (6)	0.0020 (4)	−0.0084 (5)	−0.0026 (4)
C20	0.0224 (6)	0.0154 (5)	0.0178 (6)	−0.0008 (4)	−0.0053 (4)	−0.0029 (4)
C21	0.0261 (6)	0.0239 (6)	0.0218 (6)	0.0023 (5)	−0.0035 (5)	−0.0107 (5)
C22	0.0298 (7)	0.0525 (10)	0.0517 (10)	0.0160 (7)	−0.0183 (7)	−0.0301 (8)
C23	0.0271 (7)	0.0285 (7)	0.0343 (7)	0.0102 (5)	−0.0137 (6)	−0.0062 (6)
C24	0.0272 (6)	0.0243 (6)	0.0260 (7)	0.0052 (5)	−0.0054 (5)	−0.0065 (5)

### Geometric parameters (Å, º)

**Table:**

O1—C20	1.3478 (14)	C11—C12	1.4099 (16)
O1—C21	1.4513 (15)	C12—C13	1.3862 (17)
O2—C20	1.2127 (15)	C12—H12A	0.9500
N1—C1	1.3837 (15)	C13—H13A	0.9500
N1—C7	1.4011 (14)	C14—C15	1.3904 (17)
N1—C14	1.4404 (14)	C14—C19	1.3907 (16)
N2—C7	1.3238 (15)	C15—C16	1.3897 (17)
N2—C6	1.3866 (15)	C15—H15A	0.9500
N3—C11	1.3829 (16)	C16—C17	1.3908 (19)
N3—C24	1.4489 (17)	C16—H16A	0.9500
N3—C23	1.4500 (17)	C17—C18	1.3882 (19)
C1—C2	1.3937 (16)	C17—H17A	0.9500
C1—C6	1.4070 (16)	C18—C19	1.3923 (17)
C2—C3	1.3813 (17)	C18—H18A	0.9500
C2—H2A	0.9500	C19—H19A	0.9500
C3—C4	1.4115 (16)	C21—C22	1.491 (2)
C3—H3A	0.9500	C21—H21A	0.9900
C4—C5	1.3909 (16)	C21—H21B	0.9900
C4—C20	1.4805 (17)	C22—H22A	0.9800
C5—C6	1.3908 (17)	C22—H22B	0.9800
C5—H5A	0.9500	C22—H22C	0.9800
C7—C8	1.4653 (16)	C23—H23A	0.9800
C8—C13	1.3995 (17)	C23—H23B	0.9800
C8—C9	1.4069 (16)	C23—H23C	0.9800
C9—C10	1.3802 (17)	C24—H24A	0.9800
C9—H9A	0.9500	C24—H24B	0.9800
C10—C11	1.4087 (18)	C24—H24C	0.9800
C10—H10A	0.9500		
			
C20—O1—C21	115.44 (10)	C8—C13—H13A	119.2
C1—N1—C7	106.59 (9)	C15—C14—C19	121.28 (11)
C1—N1—C14	122.51 (9)	C15—C14—N1	120.01 (10)
C7—N1—C14	130.52 (10)	C19—C14—N1	118.68 (11)
C7—N2—C6	105.79 (10)	C16—C15—C14	118.87 (11)
C11—N3—C24	119.19 (11)	C16—C15—H15A	120.6
C11—N3—C23	119.82 (11)	C14—C15—H15A	120.6
C24—N3—C23	117.76 (11)	C15—C16—C17	120.50 (12)
N1—C1—C2	131.70 (11)	C15—C16—H16A	119.8
N1—C1—C6	105.58 (10)	C17—C16—H16A	119.8
C2—C1—C6	122.72 (11)	C18—C17—C16	120.03 (12)
C3—C2—C1	116.81 (11)	C18—C17—H17A	120.0
C3—C2—H2A	121.6	C16—C17—H17A	120.0
C1—C2—H2A	121.6	C17—C18—C19	120.16 (12)
C2—C3—C4	121.33 (11)	C17—C18—H18A	119.9
C2—C3—H3A	119.3	C19—C18—H18A	119.9
C4—C3—H3A	119.3	C14—C19—C18	119.14 (12)
C5—C4—C3	121.25 (11)	C14—C19—H19A	120.4
C5—C4—C20	117.45 (10)	C18—C19—H19A	120.4
C3—C4—C20	121.30 (11)	O2—C20—O1	122.55 (11)
C6—C5—C4	118.08 (10)	O2—C20—C4	124.51 (11)
C6—C5—H5A	121.0	O1—C20—C4	112.95 (10)
C4—C5—H5A	121.0	O1—C21—C22	107.42 (11)
N2—C6—C5	130.04 (11)	O1—C21—H21A	110.2
N2—C6—C1	110.18 (10)	C22—C21—H21A	110.2
C5—C6—C1	119.77 (11)	O1—C21—H21B	110.2
N2—C7—N1	111.84 (10)	C22—C21—H21B	110.2
N2—C7—C8	122.37 (10)	H21A—C21—H21B	108.5
N1—C7—C8	125.72 (11)	C21—C22—H22A	109.5
C13—C8—C9	116.88 (11)	C21—C22—H22B	109.5
C13—C8—C7	125.18 (11)	H22A—C22—H22B	109.5
C9—C8—C7	117.88 (11)	C21—C22—H22C	109.5
C10—C9—C8	122.03 (12)	H22A—C22—H22C	109.5
C10—C9—H9A	119.0	H22B—C22—H22C	109.5
C8—C9—H9A	119.0	N3—C23—H23A	109.5
C9—C10—C11	121.03 (11)	N3—C23—H23B	109.5
C9—C10—H10A	119.5	H23A—C23—H23B	109.5
C11—C10—H10A	119.5	N3—C23—H23C	109.5
N3—C11—C10	121.81 (11)	H23A—C23—H23C	109.5
N3—C11—C12	121.07 (11)	H23B—C23—H23C	109.5
C10—C11—C12	117.11 (11)	N3—C24—H24A	109.5
C13—C12—C11	121.30 (11)	N3—C24—H24B	109.5
C13—C12—H12A	119.4	H24A—C24—H24B	109.5
C11—C12—H12A	119.4	N3—C24—H24C	109.5
C12—C13—C8	121.63 (11)	H24A—C24—H24C	109.5
C12—C13—H13A	119.2	H24B—C24—H24C	109.5
			
C7—N1—C1—C2	−179.19 (12)	C8—C9—C10—C11	−0.05 (19)
C14—N1—C1—C2	7.2 (2)	C24—N3—C11—C10	−166.84 (12)
C7—N1—C1—C6	1.38 (12)	C23—N3—C11—C10	−7.59 (19)
C14—N1—C1—C6	−172.24 (10)	C24—N3—C11—C12	13.84 (18)
N1—C1—C2—C3	−178.49 (12)	C23—N3—C11—C12	173.09 (12)
C6—C1—C2—C3	0.86 (18)	C9—C10—C11—N3	179.62 (12)
C1—C2—C3—C4	0.58 (18)	C9—C10—C11—C12	−1.04 (18)
C2—C3—C4—C5	−1.15 (18)	N3—C11—C12—C13	−179.78 (12)
C2—C3—C4—C20	179.44 (11)	C10—C11—C12—C13	0.87 (18)
C3—C4—C5—C6	0.24 (17)	C11—C12—C13—C8	0.40 (19)
C20—C4—C5—C6	179.67 (10)	C9—C8—C13—C12	−1.47 (18)
C7—N2—C6—C5	−178.08 (12)	C7—C8—C13—C12	−178.60 (11)
C7—N2—C6—C1	1.17 (13)	C1—N1—C14—C15	108.78 (13)
C4—C5—C6—N2	−179.65 (11)	C7—N1—C14—C15	−63.17 (17)
C4—C5—C6—C1	1.16 (17)	C1—N1—C14—C19	−69.23 (15)
N1—C1—C6—N2	−1.61 (13)	C7—N1—C14—C19	118.82 (13)
C2—C1—C6—N2	178.89 (11)	C19—C14—C15—C16	−1.54 (18)
N1—C1—C6—C5	177.73 (10)	N1—C14—C15—C16	−179.49 (11)
C2—C1—C6—C5	−1.76 (18)	C14—C15—C16—C17	0.87 (19)
C6—N2—C7—N1	−0.27 (13)	C15—C16—C17—C18	0.43 (19)
C6—N2—C7—C8	−177.42 (10)	C16—C17—C18—C19	−1.10 (19)
C1—N1—C7—N2	−0.73 (13)	C15—C14—C19—C18	0.88 (18)
C14—N1—C7—N2	172.19 (11)	N1—C14—C19—C18	178.86 (11)
C1—N1—C7—C8	176.31 (11)	C17—C18—C19—C14	0.45 (19)
C14—N1—C7—C8	−10.77 (19)	C21—O1—C20—O2	−3.58 (17)
N2—C7—C8—C13	169.33 (11)	C21—O1—C20—C4	176.91 (10)
N1—C7—C8—C13	−7.41 (19)	C5—C4—C20—O2	−1.07 (18)
N2—C7—C8—C9	−7.78 (17)	C3—C4—C20—O2	178.36 (12)
N1—C7—C8—C9	175.48 (11)	C5—C4—C20—O1	178.43 (10)
C13—C8—C9—C10	1.30 (18)	C3—C4—C20—O1	−2.14 (16)
C7—C8—C9—C10	178.65 (11)	C20—O1—C21—C22	177.00 (12)

### Hydrogen-bond geometry (Å, º)

**Table:**

*D*—H···*A*	*D*—H	H···*A*	*D*···*A*	*D*—H···*A*
C15—H15*A*···N2^i^	0.95	2.57	3.4868 (16)	164
C18—H18*A*···O2^ii^	0.95	2.50	3.2217 (17)	133
C19—H19*A*···O2^iii^	0.95	2.38	3.3209 (16)	170

Symmetry codes: (i) −*x*+2, −*y*+1, −*z*+1; (ii) *x*, *y*, *z*+1; (iii) −*x*+2, −*y*, −*z*+1.
